# Correction: A phosphoswitch at acinus-serine^437^ controls autophagic responses to cadmium exposure and neurodegenerative stress

**DOI:** 10.7554/eLife.108297

**Published:** 2025-07-09

**Authors:** Nilay Nandi, Zuhair Zaidi, Charles Tracy, Helmut Krämer

**Keywords:** *D. melanogaster*

Nandi N, Zaidi Z, Tracy C, Krämer H. 2022. A phosphoswitch at acinus-serine^437^ controls autophagic responses to cadmium exposure and neurodegenerative stress. *eLife* **11**:e72169. doi: 10.7554/eLife.72169.

Published 17 January 2022

We were notified via PubPeer of a duplication in Figure 1. Upon examining other figures for similar issues, we found a duplication in Figure 5.

The inclusion of these duplicates were regrettable errors. We presume the duplications happened while choosing the images from the folders with the original data.

The image of the eye of the GMR-Gal4, UAS-Nil-RNAi fly in Figure 1 panel N was correctly attributed but was duplicated in panel M. We since retrieved the image corresponding to the GMR-Gal4, UAS-CG15035-RNAi eye and replaced the duplicated image in panel M.

The image from the Cd2+^2+^-treated AcnS437A^S437A^ eye disc in Figure 5 panel H was duplicated in Figure 5 panel F. We have retrieved the image for the untreated AcnS437A^S437A^ eye disc and replaced the duplicated image.

The corrected Figure 1 is shown here:

**Figure 1:**
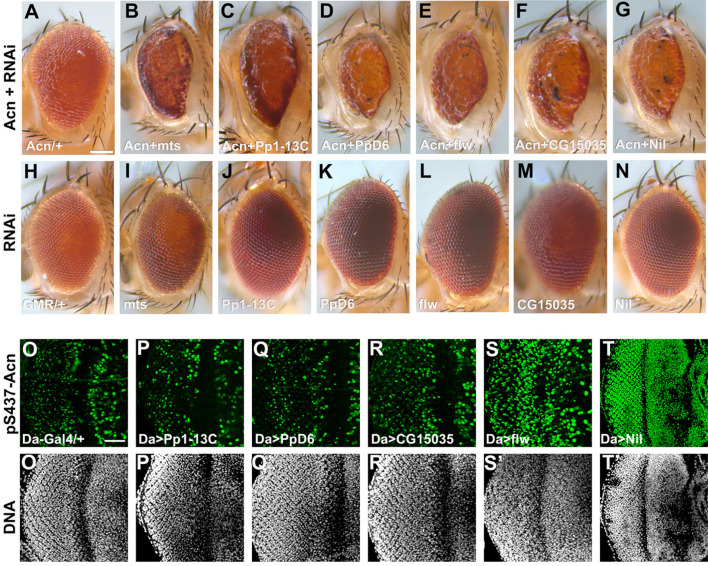


The originally published Figure 1 is shown for reference:

**Figure 2:**
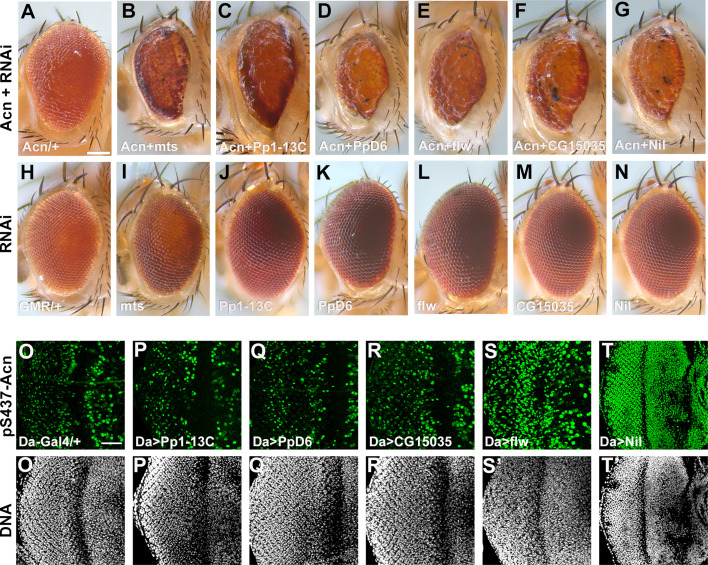


The corrected Figure 5 is shown here:

**Figure 3:**
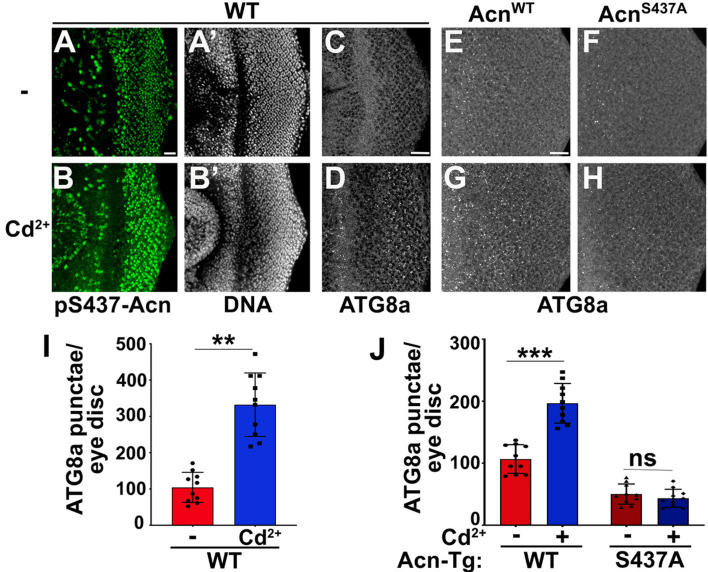


The originally published Figure 5 is shown for reference:

**Figure 4:**
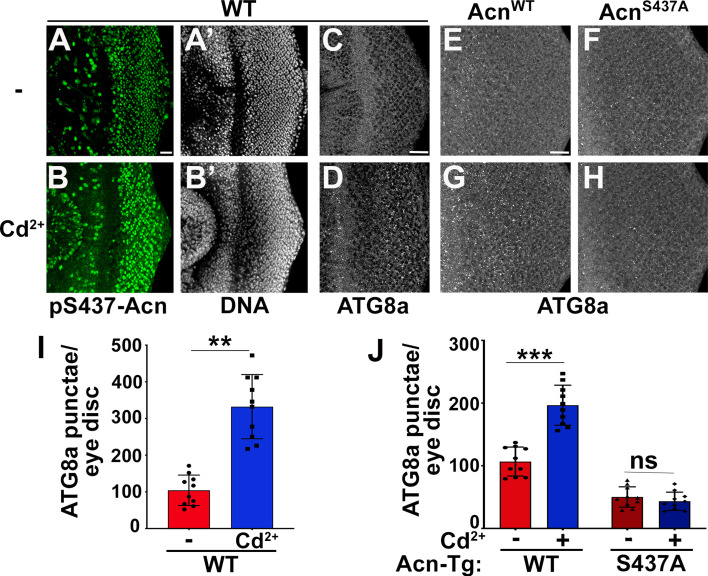


The article has been corrected accordingly.

