# Supplementary figures and images for: A phosphoswitch at acinus-serine437 controls autophagic responses to cadmium exposure and neurodegenerative stress
Source: eLife. 2022 Jan 17;11:e72169. doi: 10.7554/eLife.72169 (PMC8794470; doi:10.7554/eLife.72169)

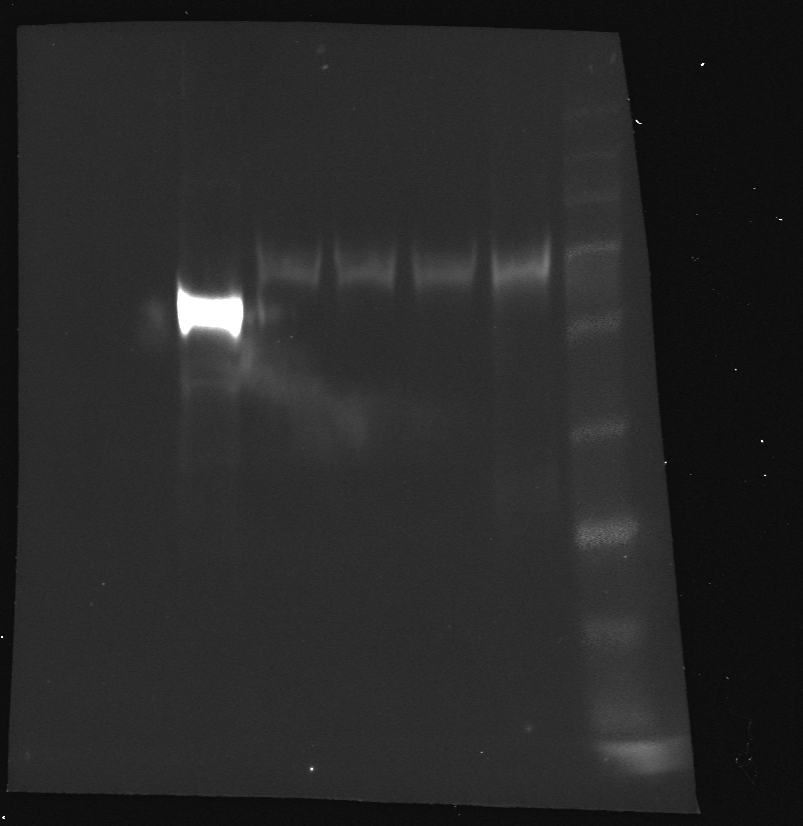

Supplement: Figure 2—figure supplement 2—source data 1. — Western blot analysis using anti-Ty1 and anti-Hook antibodies in lysates from adult males of nilTy1G4 and appropriate control. The parts of the raw image used in Figure 2—figure supplement 2O were marked with box. [file elife-72169-fig2-figsupp2-data1.zip › Figure 2 -Supplement 2 source data/Raw data Ty1 converted 16bit.tif]

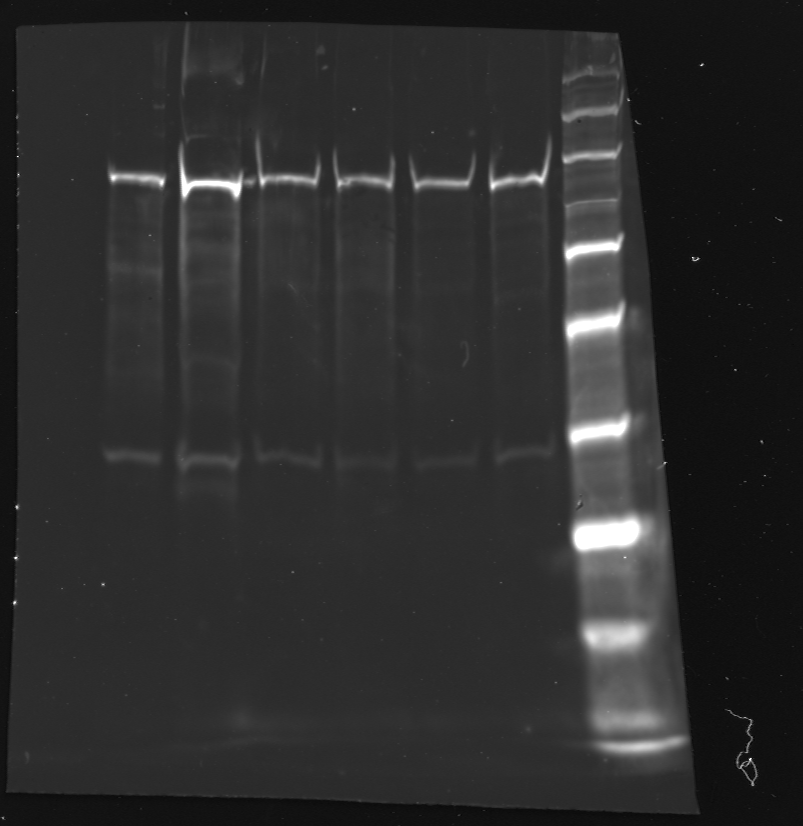

Supplement: Figure 2—figure supplement 2—source data 1. — Western blot analysis using anti-Ty1 and anti-Hook antibodies in lysates from adult males of nilTy1G4 and appropriate control. The parts of the raw image used in Figure 2—figure supplement 2O were marked with box. [file elife-72169-fig2-figsupp2-data1.zip › Figure 2 -Supplement 2 source data/Raw data Hook converted 16bit.tif]

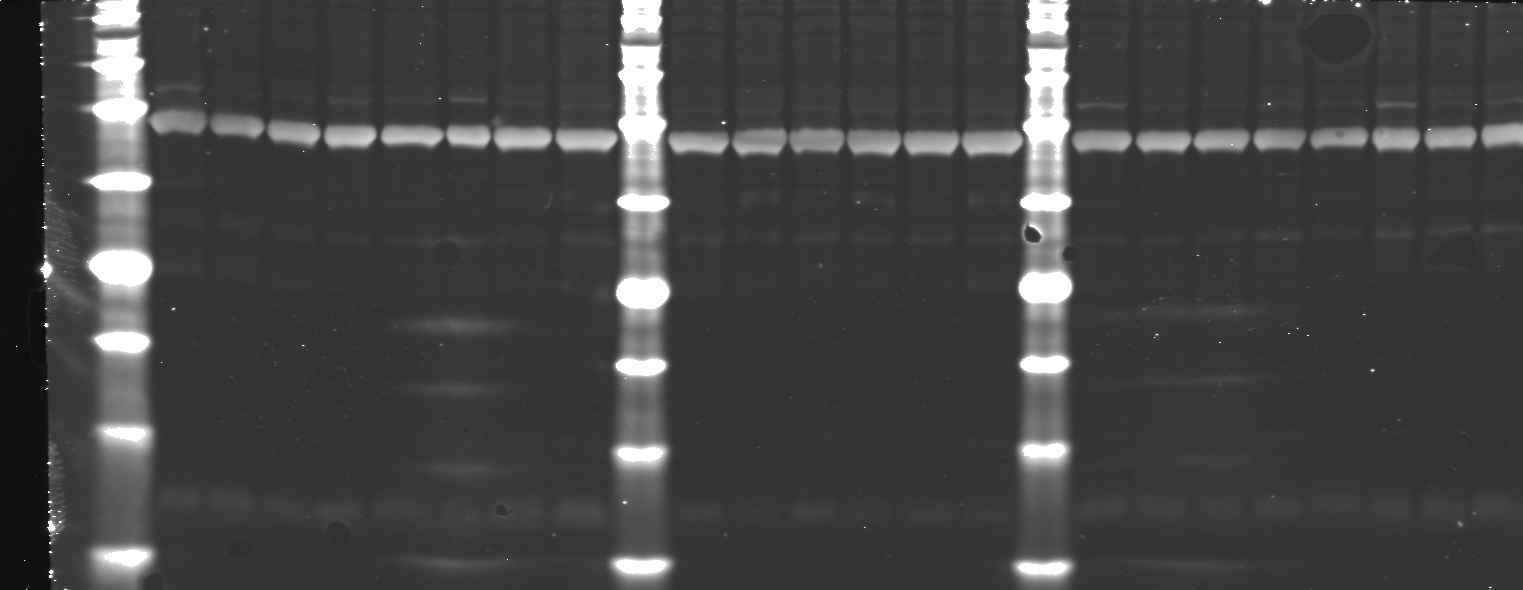

Supplement: Figure 3—source data 1. — Western blot analysis using anti-ATG8a and anti-actin antibodies in lysates from adult heads of w1118 and nil1. Boxes mark the parts of the raw image used in Figure 3A. [file elife-72169-fig3-data1.zip › Figure 3A source data /Raw data Actin converted 16bit.tif]

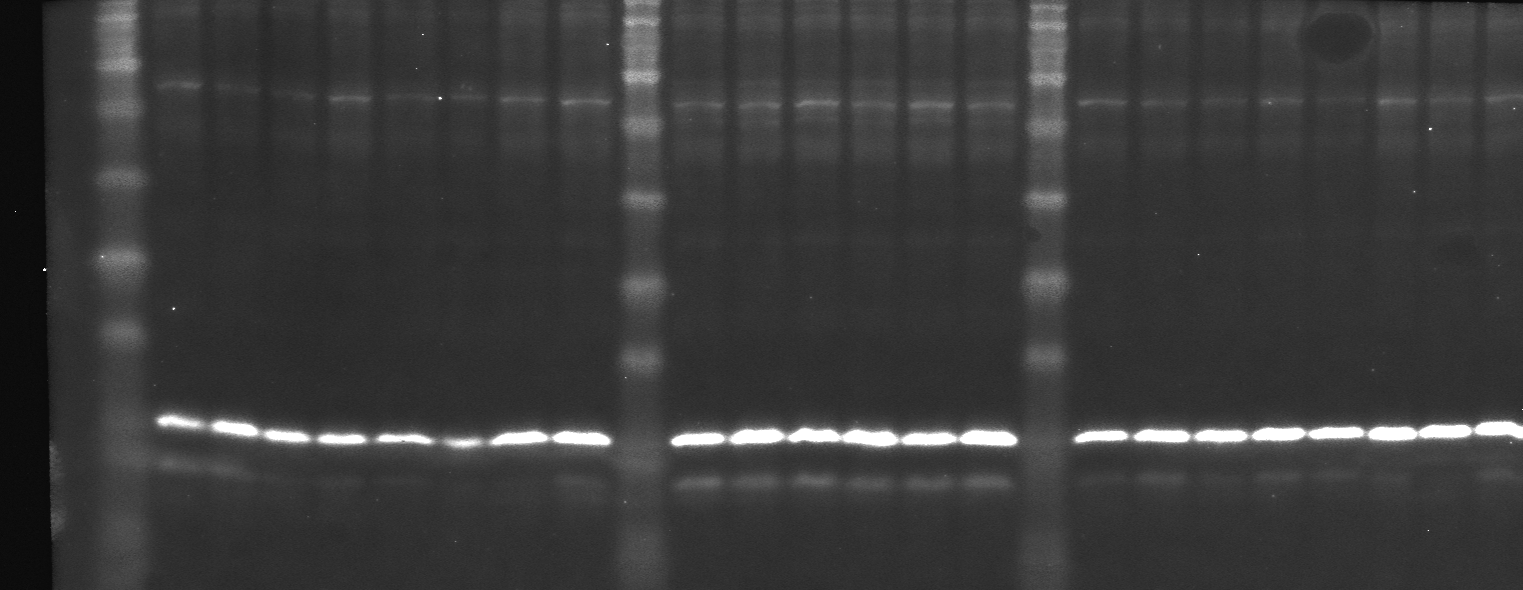

Supplement: Figure 3—source data 1. — Western blot analysis using anti-ATG8a and anti-actin antibodies in lysates from adult heads of w1118 and nil1. Boxes mark the parts of the raw image used in Figure 3A. [file elife-72169-fig3-data1.zip › Figure 3A source data /Raw data ATG8a converted 16bit.tif]
