# Supplementary material for: A phosphoswitch at acinus-serine437 controls autophagic responses to cadmium exposure and neurodegenerative stress
Source: eLife. 2022 Jan 17;11:e72169. doi: 10.7554/eLife.72169 (PMC8794470; doi:10.7554/eLife.72169)
Supplement: Supplementary file 1. — All flies were raised at 28°C. Scores to calculate average roughness: normal = 1; mild = 2; rough = 3; strongly rough = 4. Positive or negative numbers indicate suppression and enhancement, respectively. Green or red colors highlight UAS-transgenes with more than 45% suppression or enhancement. Numbers in parenthesis indicated stock numbers of the Bloomington Drosophila stock center. [file elife-72169-supp1.docx]

| **GMR-Gal4 driven transgenes** | **Normal** | **Mild Errors** | **Rough Eyes** | **Severely Rough** | **Avg. Score** | **n = flies scored** | Enhancement or Suppression |
| --- | --- | --- | --- | --- | --- | --- | --- |
| Acinus (Acn) | 6 | 5 | 3 | 12 | 2.8 | 26 | N/A |
| GMR-Gal4 | 56 | 10 | 0 | 0 | 1.2 | 66 | N/A |
| Acn + Alphabet (31398) | 12 | 17 | 12 | 26 | 2.8 | 67 | 0% |
| Alphabet (31398) | 31 | 21 | 0 | 0 | 1.4 | 52 |  |
| Acn + Alphabet (40873) | 1 | 6 | 30 | 53 | 3.2 | 90 | 33.24% |
| Alphabet (40873) | 38 | 33 | 20 | 3 | 1.9 | 96 |  |
| Acn+ CG6036 (65115) | **3** | **4** | **3** | **27** | **3.5** | **37** | **58.77%** |
| CG6036 (65115) | 42 | 5 | 0 | 0 | 1.1 | 47 |  |
| Acn + CG6036 (105568) | 3 | 23 | 5 | 24 | 2.9 | 55 | 5.56% |
| CG6036 (105568) | 15 | 9 | 12 | 18 | 1.8 | 54 |  |
| Acn + CG7115 (60015) | 8 | 10 | 8 | 15 | 2.7 | 41 | -5.39% |
| CG7115 (60015) | 47 | 20 | 0 | 0 | 1.3 | 67 |  |
| Acn + CG7115 (103354) | 5 | 22 | 2 | 17 | 3.0 | 46 | 11.11% |
| CG7115 (103354) | 23 | 23 | 10 | 0 | 1.8 | 56 |  |
| Acn + CG10376 (41907) | 15 | 13 | 12 | 5 | 2.2 | 45 | -58.56% |
| CG10376 (41907) | 64 | 8 | 0 | 0 | 1.1 | 72 |  |
| Acn + CG10376 (57036) | 3 | 6 | 0 | 26 | 3.4 | 35 | 25% |
| CG10376 (57036) | 16 | 7 | 12 | 0 | 2.4 | 31 |  |
| Acn + CG10417 (39051) | 16 | 27 | 3 | 29 | 2.3 | 75 | -41.96% |
| CG10417 (39051) | 90 | 13 | 0 | 0 | 1.1 | 103 |  |
| Acn + CG12091 (40936) | 23 | 21 | 42 | 0 | 2.7 | 86 | 7.83% |
| CG12091 (40936) | 74 | 13 | 0 | 0 | 1.2 | 87 |  |
| Acn + CG12091 (105722) | 5 | 8 | 0 | 20 | 3.1 | 33 | 17.34% |
| CG12091 (105722) | 42 | 25 | 5 | 0 | 1.7 | 70 |  |
| Acn + CG15035 (60404) | 1 | 5 | 0 | 28 | 3.6 | 34 | 74.95% |
| CG15035 (60404) | 59 | 6 | 0 | 0 | 1.1 | 65 |  |
| Acn + CG17598 (38345) | 24 | 23 | 0 | 24 | 2.3 | 71 | -44.76% |
| CG17598 (38345) | 83 | 5 | 0 | 0 | 1.1 | 88 |  |
| Acn + CG17746 (38347) | 6 | 22 | 0 | 18 | 2.7 | 46 | -14.56% |
| CG17746 (38347) | 74 | 3 | 0 | 0 | 1.03 | 77 |  |
| Acn + CG17746 (105177) | 9 | 16 | 0 | 7 | 2.2 | 32 | -50% |
| CG17746 (105177) | 70 | 3 | 3 | 0 | 1.2 | 73 |  |
| Acn + Fig (65056) | 6 | 8 | 3 | 12 | 2.7 | 39 | -7.55% |
| Fig (65056) | 59 | 4 | 0 | 0 | 1.1 | 63 |  |
| Acn + Mppe (57773) | 6 | 5 | 11 | 25 | 3.2 | 47 | 34.68% |
| Mppe (57773) | 44 | 1 | 0 | 0 | 1.0 | 45 |  |
| Acn + Mppe (5773) | 6 | 5 | 11 | 25 | 3.2 | 47 | 36.36% |
| Mppe (57773) | 44 | 1 | 0 | 0 | 1.1 | 45 |  |
| Acn + Phlpp (57349) | 14 | 16 | 4 | 39 | 3.0 | 73 | 16.51% |
| Phlpp (57349) | 58 | 6 | 0 | 0 | 1.1 | 64 |  |
| Acn + Phlpp (57399) | 14 | 16 | 4 | 39 | 2.9 | 73 | 9.09% |
| Phlpp (57399) | 58 | 6 | 0 | 0 | 1.1 | 64 |  |
| Acn + Pp2C1 (40827) | 10 | 12 | 5 | 38 | 3.1 | 65 | 27.27% |
| Pp2C1 (40827) | 41 | 7 | 0 | 0 | 1.1 | 48 |  |
| Acn + Ppm1 (41987) | 5 | 1 | 2 | 30 | 3.5 | 38 | 33.33% |
| Ppm1 (41987) | 17 | 18 | 20 | 0 | 2.1 | 55 |  |
| Acn + Pgam5 (33346) | 6 | 12 | 5 | 53 | 3.4 | 76 | 30.53% |
| Pgam5 (33346) | 22 | 17 | 21 | 0 | 1.9 | 60 |  |
| Acn + Pgam5-2 (57006) | 6 | 4 | 18 | 12 | 2.8 | 40 | 0% |
| Pgam5-2 (57006) | 44 | 15 | 9 | 0 | 1.5 | 68 |  |
| Acn + Ssu72 (57018) | 0 | 16 | 2 | 11 | 2.8 | 29 | 0% |
| Ssu72 (57018) | 25 | 5 | 0 | 0 | 1.2 | 30 |  |
| Acn + CanA1 (25850) | 19 | 6 | 0 | 45 | 3.0 | 70 | 20.39% |
| CanA1 (25850) | 65 | 2 | 0 | 0 | 1.0 | 67 |  |
| Acn + CG11597 (61988) | 13 | 10 | 0 | 8 | 2.5 | 31 | 25% |
| CG11597 (61988) | 49 | 7 | 0 | 0 | 1.1 | 56 |  |
| Acn + CG11597 (57047) | 18 | 3 | 0 | 24 | 2.9 | 45 | 8.18% |
| CG11597 (57047) | 64 | 6 | 0 | 0 | 1.1 | 70 |  |
| Acn + CG11597 (43175) | 18 | 0 | 0 | 41 | 3.1 | 59 | 18.30% |
| CG11597 (43175) | 45 | 35 | 0 | 0 | 1.5 | 75 |  |
| Acn + Flw (38336) | 24 | 12 | 0 | 45 | 2.8 | 81 | 0.91% |
| Flw (38336) | 65 | 6 | 0 | 0 | 1.1 | 71 |  |
| Acn + Flw (57022) | 5 | 1 | 0 | 24 | 3.6 | 34 | 68.91% |
| Flw (57022) | 39 | 5 | 0 | 0 | 1.1 | 44 |  |
| Acn + Mts (27723) | 0 | 0 | 2 | 64 | 4.0 | 66 | 98.33% |
| Mts (27723) | 37 | 7 | 3 | 0 | 1.2 | 50 |  |
| Acn + Mts (60342) | 11 | 13 | 2 | 9 | 2.3 | 35 | -33.42% |
| Mts (60342) | 15 | 25 | 0 | 0 | 1.6 | 40 |  |
| Acn + Mts (57034) | 0 | 0 | 0 | 2 | 4.0 | 2 | 42.86% |
| Mts (57034) | 0 | 0 | 0 | 1 | 4 | 1 |  |
| Acn + Pp1-13C (32465) | 3 | 4 | 0 | 48 | 3.7 | 55 | 66.92% |
| Pp1-13C (32465) | 30 | 15 | 0 | 0 | 1.3 | 45 |  |
| Acn + Pp1-13C (107770) | 6 | 17 | 13 | 18 | 2.8 | 54 | 0% |
| Pp1-13C (107770) | 55 | 20 | 17 | 5 | 1.8 | 92 |  |
| Acn + Pp1-87B (32414) | 67 | 0 | 0 | 0 | 4.0 | 67 | 33.33% |
| Pp1-87B (32414) | 0 | 0 | 3 | 27 | 3.6 | 30 |  |
| Acn + Pp1α-96A (40906) | 29 | 8 | 0 | 14 | 2.1 | 48 | -61.65% |
| Pp1α-96A (40906) | 27 | 6 | 0 | 0 | 1.1 | 33 |  |
| Acn + Pp1α-96A (42641) | 14 | 10 | 22 | 0 | 2.4 | 46 | -22.22% |
| Pp1α-96A (42641) | 10 | 3 | 0 | 6 | 1.8 | 19 |  |
| Acn + Pp1-Y1 (58098) | 13 | 13 | 9 | 6 | 2.2 | 41 | -54.95% |
| Pp1-Y1 (58098) | 64 | 8 | 0 | 0 | 1.1 | 72 |  |
| Acn + Pp1-Y1 (65924) | 8 | 2 | 0 | 35 | 3.4 | 45 | 33.33% |
| Pp1-Y1 (65924) | 15 | 18 | 4 | 0 | 1.8 | 37 |  |
| Acn + Pp1-Y2 (57236) | 18 | 10 | 0 | 23 | 2.6 | 51 | -19.69% |
| Pp1-Y2 (57236) | 47 | 18 | 0 | 0 | 1.3 | 65 |  |
| Acn + Pp2B-14D (25929) | 31 | 23 | 6 | 13 | 2.0 | 73 | -72.48% |
| Pp2B-14D (25929) | 48 | 5 | 0 | 0 | 1.1 | 53 |  |
| Acn + Pp4-19C (27726) | 18 | 17 | 3 | 19 | 2.4 | 57 | -36.76% |
| Pp4-19C (27726) | 62 | 6 | 0 | 0 | 1.1 | 68 |  |
| Acn + Pp4-19C (57823) | 0 | 3 | 19 | 54 | 3.3 | 76 | 32.86% |
| Pp4-19C (57823) | 15 | 10 | 27 | 0 | 2.1 | 52 |  |
| Acn + PpD3 (42794) | 8 | 8 | 7 | 17 | 2.8 | 40 | 2.29% |
| PpD3 (42794) | 38 | 4 | 0 | 0 | 1.1 | 42 |  |
| Acn + PpD6 (62849) | 3 | 3 | 1 | 26 | 3.5 | 33 | 48.27% |
| PpD6 (62849) | 28 | 25 | 0 | 0 | 1.5 | 53 |  |
| Acn + PpN58A (57402) | 16 | 20 | 2 | 36 | 2.8 | 74 | -1.28% |
| PpN58A (57402) | 40 | 53 | 0 | 0 | 1.6 | 93 |  |
| Acn + PpV (36064) | 3 | 3 | 2 | 25 | 3.5 | 33 | 48.90% |
| PpV (36064) | 32 | 18 | 2 | 0 | 1.4 | 52 |  |
| Acn + PpV (57765) | 23 | 26 | 6 | 12 | 2.1 | 67 | -56.00% |
| PpV (57765) | 57 | 19 | 0 | 0 | 1.3 | 76 |  |
| Acn + PpY-55A (57519) | 33 | 19 | 0 | 0 | 1.4 | 52 | -113.89% |
| PpY-55A (57519) | 37 | 13 | 0 | 0 | 1.3 | 50 |  |
| Acn + RdgC (60076) | 23 | 28 | 1 | 29 | 2.5 | 71 | -17.44% |
| RdgC (60076) | 14 | 36 | 0 | 0 | 1.7 | 50 |  |
|  |  |  |  |  |  |  |  |
|  |  |  |  |  |  |  |  |

Supplementary File 1. Effect of knockdown of different phosphatases on eye roughness in a Acn gain-of-function model.

All flies were raised at 28°C.

Scores to calculate average roughness: normal = 1; mild = 2; rough = 3; strongly rough = 4.

(1) Positive or negative numbers indicate suppression and enhancement, respectively.

(2) Green or red colors highlight UAS-transgenes with more than 45% suppression or enhancement.

(3) Numbers in parenthesis indicated stock numbers at the Bloomington Drosophila stock center.
