# Supplementary material for: A phosphoswitch at acinus-serine437 controls autophagic responses to cadmium exposure and neurodegenerative stress
Source: eLife. 2022 Jan 17;11:e72169. doi: 10.7554/eLife.72169 (PMC8794470; doi:10.7554/eLife.72169)
Supplement: Supplementary file 2. — All flies were raised at 28°C. Scores to calculate depigmentation and roughness: Score 1: No depigmentation and no roughness. Score 2: Mild depigmentation and no roughness. Score 3: Moderate depigmentation and roughness. Score 4: Extreme depigmentation and roughness. [file elife-72169-supp2.docx]

Supplementary File 2. Effect of loss and gain of phosphatase activity on eye pigmentation in a Drosophila Huntington's model.

| Genotype | Scores for Depigmented Fly Eye | | | |
| --- | --- | --- | --- | --- |
|  | 1 | 2 | 3 | 4 |
| GMR>Q93 | 0 | 8 | 56 | 39 |
| GMR>Q93+Nil RNAi | 5 | 72 | 17 | 0 |
| GMR>Q93+hPPM1B | 0 | 17 | 45 | 31 |
| GMR/+ | 93 | 0 | 0 | 0 |
| GMR>Nil RNAi | 97 | 0 | 0 | 0 |
| GMR>hPPM1B | 72 | 0 | 0 | 0 |

All flies were raised at 28°C. Scores to calculate Depigmentation and Roughness:

Score 1: No depigmentation and no roughness

Score 2: Mild depigmentation and no roughness

Score 3: Moderate depigmentation and roughness

Score 4: Extreme depigmentation and roughness
