# Supplementary material for: A phosphoswitch at acinus-serine437 controls autophagic responses to cadmium exposure and neurodegenerative stress
Source: eLife. 2022 Jan 17;11:e72169. doi: 10.7554/eLife.72169 (PMC8794470; doi:10.7554/eLife.72169)
Supplement: Supplementary file 3. — The table list relevant genotype for flies used in each figure. [file elife-72169-supp3.docx]

Supplementary File 3. Genotypes of Flies Used for Each Figure

| **Figure 1** | **Genotype** |
| --- | --- |
| 1 A | *w^*^* ; GMR-Gal4, P[w+, UAS-Acn^WT^] / CyO; + / + |
| 1 B | *w^*^* ; GMR-Gal4, P[w+, UAS-Acn^WT^] / +; UAS-Mts TRIP (BS 27723) / + |
| 1 C | *w^*^* ; GMR-Gal4, P[w+, UAS-Acn^WT^] / +; UAS-Pp1-13C TRIP (BS 32465) / + |
| 1 D | *w^*^* ; GMR-Gal4, P[w+, UAS-Acn^WT^] / UAS-PpD6 TRIP (BS 62849); + / + |
| 1 E | *w^*^* ; GMR-Gal4, P[w+, UAS-Acn^WT^] / +; UAS-Flw TRIP (BS 38336) / + |
| 1 F | *w^*^* ; GMR-Gal4, P[w+, UAS-Acn^WT^] / UAS-CG15035 TRIP (BS 60404); + / + |
| 1 G | *w^*^* ; GMR-Gal4, P[w+, UAS-Acn^WT^] / UAS-Nil TRIP (BS 65115); + / + |
| 1 H | *w^*^* ; GMR*-*Gal4 / +; *+* / + |
| 1 I | *w^*^* ; GMR*-*Gal4 / +; UAS-Mts TRIP (BS 27723) / + |
| 1 J | *w^*^* ; GMR*-*Gal4 / +; UAS-Pp1-13C TRIP (BS 32465) / + |
| 1 K | *w^*^* ; GMR*-*Gal4 / UAS-PpD6 TRIP (BS 62849); + / + |
| 1 L | *w^*^* ; GMR*-*Gal4 / +; UAS-Flw TRIP (BS 38336) / + |
| 1 M | *w^*^* ; GMR*-*Gal4 / UAS-CG15035 TRIP (BS 60404); + / + |
| 1 N | *w^*^* ; GMR*-*Gal4 / UAS-Nil TRIP (BS 65115); + / + |
| 1 O | *w^*^* ; CyO / +; Da*-*Gal4 / + |
| 1 P | *w^*^* ; CyO / +; Da*-*Gal4 / UAS-Pp1-13C TRIP (BS 32465) |
| 1 Q | *w^*^* ; CyO / UAS-PpD6 TRIP (BS 62849); Da*-*Gal4 / + |
| 1 R | *w^*^* ; CyO / UAS-CG15035 TRIP (BS 60404); Da*-*Gal4 / + |
| 1 S | *w^*^* ; CyO / +; Da*-*Gal4 / UAS-Flw TRIP (BS 38336) |
| 1 T | *w^*^* ; CyO / UAS-Nil TRIP (BS 65115); Da*-*Gal4 / + |
|  |  |
| **Figure 2** | **Genotype** |
| 2 B | *w*^1118^ |
| 2 C | *nil*^1^ |
| 2 D | *w*^1118^ |
| 2 E | *nil*^1^ |
| 2 F | *w^*^* ; GMR*-*Gal4 / +; *+* / + |
| 2 G | *nil*^1^ |
| 2 H | *w^*^* ; GMR*-*Gal4 / UAS-Nil^WT^-V5; *nil*^1^ |
| 2 I | *w^*^* ; GMR*-*Gal4 / UAS-hPPM1B-HA (BS 76916); *nil*^1^ |
| 2 J | *w^*^* ; GMR*-*Gal4 / UAS Nil^D231N^-V5; *nil*^1^ |
| 2 K | *w^*^* ; +/+; *nil*^Ty1-Gal4^ |
| 2 L | *w^*^* ; YFP^MYC^-Rab5/+; *nil*^Ty1-Gal4^ /+ |
| 2 M | *w^*^* ; +/+; *nil*^Ty1-Gal4^ |
| 2 N | *w^*^* ; +/+; *nil*^Ty1-Gal4^ |
| 2 O | *w^*^* ; +/+; *nil*^Ty1-Gal4^ |
|  |  |
| **Figure 3** | **Genotype** |
| 3 A, B | *w*^1118^ |
|  | *nil*^1^ |
| 3 C | *w*^1118^ |
| 3 D | *nil*^1^ |
| 3 E | *w*^1118^  *nil*^1^ |
| 3 F | *w*^1118^ |
| 3 G | *nil*^1^ |
| 3 H | *w*^1118^  *nil*^1^ |
| 3 I | *w*^1118^ |
| 3 J | *nil*^1^ |
| 3 K | *w*^1118^ |
| 3 L | *nil*^1^ |
| 3 M | *w*^1118^ |
| 3 N | *nil*^1^ |
| 3 O | *w*^1118^ |
| 3 P | *nil*^1^ |
| 3 Q | *w*^1118^  *nil*^1^ |
| 3 R | *w*^1118^  *nil*^1^ |
|  |  |
| **Figure 4** | **Genotype** |
| 4 A | *w*^1118^ |
| 4 B | *nil*^1^ |
| 4 C | *w*^1118^ |
| 4 D | *nil*^1^ |
| 4 E | *w*^1118^ |
| 4 F | *nil*^1^ |
| 4 G | *w*^1118^ |
| 4 H | *p35*^20C^ |
| 4 I | *nil*^1^ |
| 4 J | *p35*^20C^ ; *nil*^1^ |
| 4 K-N | w^*^; *acn^1^* /ubi-GFP *acn^27^* ubi-GFP; P[w+, acnP-Myc-Acn^WT^] ^96F3^ |
|  |  |
| **Figure 5** | **Genotype** |
| 5 A | *w*^1118^ |
| 5 B | *w*^1118^ |
| 5 C | *w*^1118^ |
| 5 D | *w*^1118^ |
| 5 E | w^*^; *acn^1^* /ubi-GFP *acn^27^* ubi-GFP; P[w+, acnP-Myc-Acn^WT^] ^96F3^ |
| 5 F | w^*^; *acn^1^* /ubi-GFP *acn^27^* ubi-GFP; P[w+, acnP-Myc-Acn^S437A^] ^96F3^ |
| 5 G | w^*^; *acn^1^* /ubi-GFP *acn^27^* ubi-GFP; P[w+, acnP-Myc-Acn^WT^] ^96F3^ |
| 5 H | w^*^; *acn^1^* /ubi-GFP *acn^27^* ubi-GFP; P[w+, acnP-Myc-Acn^S437A^] ^96F3^ |
| 5 I | *w*^1118^ |
| 5 J | w^*^; *acn^1^* /ubi-GFP *acn^27^* ubi-GFP; P[w+, acnP-Myc-Acn^WT^] ^96F3^  w^*^; *acn^1^* /ubi-GFP *acn^27^* ubi-GFP; P[w+, acnP-Myc-Acn^S437A^] ^96F3^ |
|  |  |
| **Figure 6** | **Genotype** |
| 6 A-C | *w*^1118^  *nil*^1^ |
| 6 D | *w^*^* ; GMR*-*Gal4 / +; UAS-Htt-exon1-Q93 / + |
| 6 E | *w^*^* ; GMR*-*Gal4 / UAS-Nil TRIP (BS 65115); UAS-Htt-exon1-Q93 / + |
| 6 F | *w^*^* ; GMR*-*Gal4 / UAS-hPPM1B-HA (BS 76916); UAS-Htt-exon1-Q93 / + |
| 6 G | *w^*^* ; GMR*-*Gal4 / +; *+* / + |
| 6 H | *w^*^* ; GMR*-*Gal4 / UAS-Nil TRIP (BS 65115); + / + |
| 6 I | *w^*^* ; GMR*-*Gal4 / UAS-hPPM1B-HA (BS 76916); + / + |
| 6 J | *w^*^* ; GMR*-*Gal4 / +; *+* / + |
| 6 K | *w^*^* ; GMR*-*Gal4 / +; UAS-Htt-exon1-Q93 / + |
| 6 L | *w^*^* ; GMR*-*Gal4 / UAS-Nil TRIP (BS 65115); UAS-Htt-exon1-Q93 / + |
| 6 M | *w^*^* ; GMR*-*Gal4 / UAS-hPPM1B-HA (BS 76916); UAS-Htt-exon1-Q93 / + |
| 6 N | *w^*^* ; GMR*-*Gal4 / +; *+* / + |
|  | *w^*^* ; GMR*-*Gal4 / +; UAS-Htt-exon1-Q93 / + |
|  | *w^*^* ; GMR*-*Gal4 / UAS-Nil TRIP (BS 65115); UAS-Htt-exon1-Q93 / + |
|  | *w^*^* ; GMR*-*Gal4 / UAS-hPPM1B-HA (BS 76916); UAS-Htt-exon1-Q93 / + |
|  |  |
|  |  |
|  |  |
|  |  |
|  |  |
|  |  |
|  |  |
| **Figure 1-Fig Supplement 1** | **Genotype** |
|  | *w^*^* ; CyO / +; Da*-*Gal4 / +  *w^*^* ; CyO / +; Da*-*Gal4 / UAS-Pp1-13C TRIP (BS 32465)  *w^*^* ; CyO / UAS-PpD6 TRIP (BS 62849); Da*-*Gal4 / +  *w^*^* ; CyO / +; Da*-*Gal4 / UAS-Flw TRIP (BS 38336)  *w^*^* ; CyO / UAS-CG15035 TRIP (BS 60404); Da*-*Gal4 / +  *w^*^* ; CyO / UAS-Nil TRIP (BS 65115); Da*-*Gal4 / + |
|  |  |
| **Figure 2-Fig Supplement 1** | **Genotype** |
| 2-S1A | *w^*^* ; GMR-Gal4, P[w+, UAS-Acn^WT^] / CyO; *Sb* / + |
| 2-S1B | *w^*^* ; GMR-Gal4, P[w+, UAS-Acn^WT^] / UAS- Nil^WT^-V5; *Sb* / + |
| 2-S1C | *w^*^* ; GMR-Gal4, P[w+, UAS-Acn^WT^] / UAS-Nil^D231N^-V5; *Sb* / + |
| 2-S1D | *w^*^* ; GMR*-*Gal4 / CyO; *Sb* / + |
| 2-S1E | *w^*^* ; GMR*-*Gal4 / UAS-Nil^WT^-V5; *Sb* / + |
| 2-S1F | *w^*^* ; GMR*-*Gal4 / UAS-Nil^D231N^-V5; *Sb* / + |
| 2-S1G | *w^*^* ; GMR*-*Gal4 / CyO; *Sb* / + |
| 2-S1H | *w^*^* ; GMR*-*Gal4 / UAS- Nil^WT^-V5; *Sb* / + |
| 2-S1I | *w^*^* ; GMR*-*Gal4 / UAS- UAS-Nil^D231N^-V5; *Sb* / + |
|  |  |
| **Figure 2-Fig Supplement 2** | **Genotype** |
| 2-S2A | *y^1^w^*^*; PBac{y[+mDint2] w[+mC]=20XUAS-6XmCherry-HA}VK00018 / +, P{Wee-P.ph0}Bacc[Wee-P20]; + / TM6C, *Sb*, *Tb*^1^ |
| 2-S2B | *w^*^* ; +/+; *nil*^Ty1-Gal4^ |
| 2-S2C | *y^1^w^*^*; PBac{y[+mDint2] w[+mC]=20XUAS-6XmCherry-HA}VK00018 / +, P{Wee-P.ph0}Bacc[Wee-P20]; + / TM6C, *Sb*, *Tb*^1^ |
| 2-S2D | *w^*^* ; +/+; *nil*^Ty1-Gal4^ |
| 2-S2E | *w^*^* ; +/+; *nil*^Ty1-Gal4^ |
| 2-S2F | *w^+^* RNAi; UAS-mCD8-GFP |
| 2-S2G | *w^+^* RNAi/+; UAS-mCD8-GFP/ *nil*^Ty1-Gal4^ |
| 2-S2H | *w^+^* RNAi; UAS-mCD8-GFP |
| 2-S2I | *w^+^* RNAi/+; UAS-mCD8-GFP/ *nil*^Ty1-Gal4^ |
| 2-S2J | *w^+^* RNAi/+; UAS-mCD8-GFP/ *nil*^Ty1-Gal4^ |
| 2-S2K | *w^+^* RNAi; UAS-mCD8-GFP |
| 2-S2L | *w^+^* RNAi/+; UAS-mCD8-GFP/ *nil*^Ty1-Gal4^ |
| 2-S2M | *w^+^* RNAi; UAS-mCD8-GFP |
| 2-S2N | *w^+^* RNAi/+; UAS-mCD8-GFP/ *nil*^Ty1-Gal4^ |
| 2-S2O | *y^1^w^*^*; PBac{y[+mDint2] w[+mC]=20XUAS-6XmCherry-HA}VK00018 / +, P{Wee-P.ph0}Bacc[Wee-P20]; + / TM6C, *Sb*, *Tb*^1^  *w^*^* ; +/+; *nil*^Ty1-Gal4^ |
|  |  |
| **Figure 3-Fig Supplement 1** | **Genotype** |
| 3-S1A | *w*^1118^  *nil*^1^ |
| 3-S1B | *w*^1118^  *nil*^1^ |
|  |  |
| **Figure 5-Fig Supplement 1** | **Genotype** |
| 5-S1A | *w*^1118^  *w^*^* ; +/+; *nil*^Ty1-Gal4^  *w^*^* ; +/+; *nil*^Ty1-Gal4^ |
| 5-S1B | *w*^1118^  *w^*^* ; +/+; *nil*^Ty1-Gal4^ |
| 5-S1C | *w*^1118^ |
| 5-S1D | *w*^1118^ |
|  |  |
| **Figure 6-Fig Supplement 1** | **Genotype** |
| 6-S1A | *w*^1118^  *nil*^1^ |
| 6-S1B | *w*^1118^  *nil*^1^ |
| 6-S1C | *w*^1118^  *nil*^1^ |
