# Supplementary material for: A phosphoswitch at acinus-serine437 controls autophagic responses to cadmium exposure and neurodegenerative stress
Source: eLife. 2022 Jan 17;11:e72169. doi: 10.7554/eLife.72169 (PMC8794470; doi:10.7554/eLife.72169)
Supplement: Supplementary file 4. — The table contains sequences of DNA oligonucleotides used for this study. [file elife-72169-supp4.docx]

Supplementary File 4 - DNA oligonucleotides used

| ***nil*^1^ Mutant** |  |
| --- | --- |
| Nil gRNA1 sense | CTTCGGATGTGATGACTAGCAGCG |
| Nil gRNA1 antisense | TTTGGCGACGATCAGTAGTGTAGG |
| Nil gRNA2 sense | CTTCGGAAATGGAGGATAGCCACT |
| Nil gRNA2 antisense | TTTGTCACCGATAGGAGGTAAAGG |
|  |  |
| ***nil*^1^ PCR Confirmation** |  |
| DsRed fwd | ACTCCAAGCTGGACATCACC |
| Nil rev | TCCGCTCTGCAATTCTTTTT |
| DsRed rev | GGGTGCTTCACGTACACCTT |
| Nil fwd | CAACATTTACCTGCGGTGTG |
|  |  |
| **Nil-Ty1 tagging** |  |
| Nil gRNA3 sense | CTTCGTAGCAACAGCTGTGCATTA |
| Nil gRNA3 antisense | TTTGATTACGTGTCGACAACGATG |
| Nil gRNA4 sense | CTTCGAGTATCTGGAAATTTCTCG |
| Nil gRNA4 antisense | TTTGGCTCTTTAAAGGTCTATGAG |
|  |  |
| **Nil tagging PCR Confirmation** |  |
| G4_fwd | GCGTATAACGCGTTTGGAAT |
| Nil_3'_rev | TTGGTTTGGTCTGCATTTGA |
| G4_rev | TCGGTTTTTCTTTGGAGCAC |
| Nil_5'_fwd | CGCAACGTGGTCATATTTTG |
|  |  |
| ***acn*^S437A^ Mutant** |  |
| Acn^S437A^ gRNA1 sense | CTTCGGCTTTGCTCAGGCTGACCT |
| Acn^S437A^ gRNA1 antisense | AAACAGGTCAGCCTGAGCAAAGCC |
| Acn^S437A^ gRNA2 sense | CTTCGGGCAACGCTTGGTCCCGGT |
| Acn^S437A^ gRNA2 antisense | AAACACCGGGACCAAGCGTTGCCC |
|  |  |
| ***acn*^S437A^ PCR Confirmation** |  |
| DsRed fwd | ACTCCAAGCTGGACATCACC |
| Acn^S437A^ rev | GGTTGCTGTGGTTTCGTTTT |
| DsRed rev | GGGTGCTTCACGTACACCTT |
| Acn^S437A^ fwd | GCCAGCCTCTCAAGAAATCA |
|  |  |
| **qPCR Primers** |  |
| RP49_ qPCR_Fwd | ATCGGTTACGGATCGAACAA |
| RP49_ qPCR_Rev | GACAATCTCCTTGCGCTTCT |
| Pp1-13C_qPCR_Fwd | GGGACTACTCTGTGACCTGC |
| Pp1-13C_qPCR_Rev | AACCATCCTCGACGACTTGA |
| PpD6_qPCR_Fwd | CTGGTGCCCTGAATCTGAAC |
| PpD6_qPCR_Rev | AAAACTGCCCGTGTATGTCG |
| flw_qPCR_Fwd | CGGGTTACTGTGCGATCTTC |
| flw_qPCR_Rev | TAGCCATCCTCCACAACCTG |
| CG15035_qPCR_Fwd | GCACCCGATTTTAAGCCGAA |
| CG15035_qPCR_Rev | CTTTACCAGATCGCACCACC |
| CG6036_qPCR_Fwd | GACTAGCAGCGAGGTTTGTG |
| CG6036_qPCR_Rev | TCAAGACTCCTTTCGGCCTT |
